# Risk Assessment for Birth Defects in Offspring of Chinese Pregnant Women

**DOI:** 10.3390/ijerph19148584

**Published:** 2022-07-14

**Authors:** Pengfei Qu, Doudou Zhao, Mingxin Yan, Danmeng Liu, Leilei Pei, Lingxia Zeng, Hong Yan, Shaonong Dang

**Affiliations:** 1 The NCH Key Laboratory of Neonatal Diseases, National Children’s Medical Center, Children’s Hospital of Fudan University, Shanghai 201102, China; xinxi3057@stu.xjtu.edu.cn; 2 Translational Medicine Center, Northwest Women’s and Children’s Hospital, No. 1616 Yanxiang Road, Xi’an 710061, China; zhaodoudou12345@stu.xjtu.edu.cn (D.Z.); liudanmeng1214@stu.xjtu.edu.cn (D.L.); 3 Department of Epidemiology and Health Statistics, School of Public Health, Xi’an Jiaotong University Health Science Center, No. 76 Yanta West Road, Xi’an 710061, China; yxjtu502@stu.xjtu.edu.cn (M.Y.); peileilei830@xjtu.edu.cn (L.P.); tjzlx@mail.xjtu.edu.cn (L.Z.); yanhonge@xjtu.edu.cn (H.Y.)

**Keywords:** birth defects, prediction model, nomogram, pregnant women, Chinese population

## Abstract

Objective: This study aimed to develop a nomogram for the risk assessment of any type of birth defect in offspring using a large birth-defect database in Northwest China. Methods: This study was based on a birth-defect survey, which included 29,204 eligible women who were pregnant between 2010 and 2013 in the Shaanxi province of Northwest China. The participants from central Shaanxi province were assigned to the training group, while the subjects from the south and north of Shaanxi province were assigned to the external validation group. The primary outcome was the occurrence of any type of birth defect in the offspring. A multivariate logistic regression model was used to establish a prediction nomogram, while the discrimination and calibration were evaluated by external validation. Results: The multivariate analyses revealed that household registration, history of miscarriages, family history of birth defects, infection, taking medicine, pesticide exposure, folic acid supplementation, and single/twin pregnancy were significant factors in the occurrence of birth defects. The area under the receiver operating characteristic curve (AUC) in the prediction model was 0.682 (95% CI 0.653 to 0.710) in the training set. The validation set showed moderate discrimination, with an AUC of 0.651 (95% CI 0.614 to 0.689). Additionally, the prediction model had a good calibration (HL χ^2^ = 8.106, *p*= 0.323). Conclusions: We developed a nomogram risk model for any type of birth defect in a Chinese population based on important modifying factors in pregnant women. This risk-prediction model could be a tool for clinicians to assess the risk of birth defects and promote health education.

## 1. Introduction

Birth defects, also known as congenital anomalies or congenital malformations, can be defined as functional or structural abnormalities in a developing fetus. Birth defects are the leading cause of early miscarriage, stillbirth, neonatal death, infant mortality, and long-term disability [1,2,3,4]. According to the World Health Organization (WHO), each year, approximately 3.2 million children worldwide are born with a congenital malformation, and about 300,000 newborns with a diagnosis of a birth defect die within the first 28 days of life [5]. As a country with a large population, China has a high total prevalence of birth defects, with an estimated rate of around 5.6% [6]. It is estimated that 800,000 to 1,200,000 children are born with malformations each year, including more than 2,500,000 cases of malformations that are visible at birth [7].

The pathogenesis of birth defects is poorly understood [1]. About 20% of birth defects are caused by simple chromosomal aberrations or gene mutations, and the remaining about 80% are caused by environmental factors or the gene–environment interaction [8]. Previous epidemiological studies have shown that mothers with lower socioeconomic status, drinking, tobacco exposure, occupational exposure, air-pollution exposure, and gestational diabetes mellitus were related to the increased risk of birth defects [9,10,11,12,13,14]. Therefore, an in-depth investigation of the potential causes of birth defects, especially environmental risk-exposure factors that could require intervention, is a key step for prenatal education and primary prevention.

Our team conducted a large population-based birth-defect survey in 2013 in Shaanxi province, Northwest China. This project is a rare study that examines the risk of birth defects. We analyzed the data from this project and identified numerous risk factors that may be associated with birth defects [15,16,17,18,19]. Pregnancy is a crucial period in fetal growth and development. Exposure to risk factors during pregnancy may affect the growth and development of the fetus. Therefore, the question of how to assess the risk of congenital malformations early and accurately is the key to the prevention of birth defects and an important scientific problem. However, the research on the predictive models of the total birth defects is limited. This study aims to develop a nomogram for the risk assessment of the total birth defects of offspring in pregnant women using a large birth-defect database in Northwest China.

## 2. Methods

### 2.1. Study Design and Population

Our study is based on a large population-based birth-defect survey conducted between August and November 2013 in Shaanxi province, Northwest China. This survey covered the central area of Shaanxi (four cities: Xi’an, Baoji, Xianyang, Weinan), south of Shaanxi (three cities: Hanzhong, Ankang, Shangluo) and north of Shaanxi (two cities: Yan’an, Yulin). The inclusion criteria were as follows: (1) The participants were women who were pregnant between August 2011 and August 2013 and gave birth before survey; (2) the participants were local residents (local household registration, or living in the local area for at least six months). The exclusion criterion was inability to communicate clearly. The maternal sociodemographic characteristics (including age, ethnicity, education, marital status, household registration, occupation) and maternal risk exposure (including lifestyle, history of pregnancy, illnesses, environment risk exposure, and folic acid supplementation) were collected from the survey. The diagnostic information on birth outcomes at local hospitals, time of diagnosis, and the types of birth defects was also collected. We also collected the occurrence of birth defects between delivery and participation in the survey. A unified questionnaire was used to conduct the investigation by trained public-health investigators. A memory-assisting strategy was used to minimize recall bias. For example, the investigator would remind participants of local crops’ pesticide application schedules to complete their pesticide exposure history, and participants were allowed to have family members to help access memories to obtain their long-term exposure history. In this survey, 82.37% of children were followed up for more than 6 months, 56.32% of children were followed up for more than 12 months, 43.69% of children were followed up for more than 18 months, and 27.80% of children were followed up for more than 24 months. A total of 30,027 women were eventually enrolled in the survey. We excluded 823 women with unclear pregnancy outcomes or missing covariates, leaving a total of 29,204 women with clear pregnancy outcomes and complete questionnaire (see supplementary Figure S1).

### 2.2. Definitions of Main Variables

Primary outcome was the occurrence of any type of birth defect in offspring, including cardiovascular system defect, musculoskeletal system defect, eye, ear, face, and neck defect, oral clefts, digestive system defect, nervous system defect, genital organ defect, respiratory system defect, urinary system defect, chromosomal abnormalities and other defects. Potential risk factors of birth defects were collected for the study participants. Considering importance and changeability of risk factors [20], fifteen predictors were included in data analysis. These included household registration (urban, rural), age (<30, ≥30), years of education (<9 years, ≥9 years), gravidity (1, ≥2), history of preterm birth (yes, no), history of miscarriages (yes, no), family history of birth defects (yes, no), infection (yes, no), taking medicine (yes, no), alcohol drinking (yes, no), tobacco exposure (yes, no), pesticide exposure (yes, no), industrial exposure (yes, no), folic acid supplementation (yes, no), and single/twin pregnancy (single, twin).

Periconceptional period was defined as the period before gestation and in early pregnancy (up to 12 weeks gestation). Age was maternal age of this pregnancy. Family history of birth defects referred to congenital disabilities of immediate relatives of the couple. Infection referred to a common cold or flu with mild viral infection at least one time during the early pregnancy. Taking medicine was defined as taking any drugs, such as antibiotics, anticancer drugs, or hormones, during early pregnancy. Alcohol drinking refers to drinking alcoholic products at least one time during the early pregnancy. Tobacco exposure was defined as active smoking (≥1 cigarette per week for 3 consecutive months) or passive smoking (≥15 min of smoke inhalation per day for 1 consecutive month) during early pregnancy. Pesticide exposure was defined as exposure to insecticide, rodenticide, herbicide, or fungicide during the periconceptional period. Industrial exposure was defined as living within 1 km of mines, paper mills, cement factories, power plants, pesticide factories, and fertilizer factories during the periconceptional period. Folic acid supplementation was defined as regularly taking folic acid only or multiple micronutrients (≥400 µg folic acid per day) in early pregnancy for a duration of more than 3 consecutive months.

### 2.3. Ethical Approval

The Human Research Ethics Committee of Xi’an Jiaotong University approved this study (no. 2012008). Written informed consent was obtained from all adult participants. 

### 2.4. Statistical Analysis

To generate nomograms and perform external verification, subjects from the center of Shaanxi province were assigned to the training group, while subjects from the south and north of Shaanxi province were assigned to the external validation group. Categorical variables were described as frequency (percentage) and the differences between groups were compared using the χ^2^ test.

We identified the factors associated with birth defects in the training group using univariate logistic regression models. Variables with statistical significance in the univariate analysis were included in the multivariate logistic regression analysis, and the forward stepwise method was used to select the variables included in the final model. A nomogram was constructed based on the multivariate logistic regression analysis results, and the selected variables were incorporated in the nomogram to predict the birth defects. 

The model performance was evaluated using the C statistic, equivalent to the receiver operating characteristic curve (ROC) area under the receiver operating characteristic curve (AUC). In addition, the calibration performance (agreement between observed and predicted frequencies of the birth defects) was assessed by Hosmer–Lemeshow (HL) χ^2^ statistics.

Statistical analysis was performed using R software (Ver 3.4.1, R Foundation for Statistical Computing, Vienna, Austria). Two-tailed analysis with *p* < 0.05 indicated that the difference was statistically significant.

## 3. Results

### 3.1. Participants’ Characteristics

In this study, 29,204 pregnant women were enrolled, including 15,723 in the development group and 13,481 in the validation group. Among the 29,204 participants, 562 women’s infants showed birth defects, including 326 cases in the development group and 236 in the validation group. Cardiovascular system defect, musculoskeletal system defect ,and eye, ear, face, and neck defect were the top three birth defects, accounting for 32.92%, 17.92%, and 12.46% of all the birth defects, respectively (see supplementary Table S1).

Table 1 shows the baseline characteristics of the pregnant women. Compared to the validation group, the women in the training group were more likely to live in urban areas, be of advanced age, have a higher education level, have a history of miscarriages, have had an infection, take medicine, have a history of exposure to tobacco, pesticides, or industrial products, and use folic acid supplementation. Finally, the training group were more likely to have less gravidity and a history of preterm birth and drink alcohol. There were no significant differences in family history of birth defects and twin pregnancy between the development and validation groups.

### 3.2. Nomogram Development

The univariate analysis between the potential predictors and birth defects in the training group is shown in Table 2. By the setting significance level to 0.05, thirteen statistically significant predictors were determined: household registration, years of education, gravidity, history of preterm birth, history of miscarriages, family history of birth defects, infection, taking medicine, tobacco exposure, pesticide exposure, industries exposure, folic acid supplementation, and single/twin pregnancy.

The multivariable logistic regression model predicting the birth defects is displayed in Table 3. The model showed that the odds of birth defects decreased with living in urban areas (OR = 0.46, 95% CI = 0.36, 0.60) and folic acid supplementation (OR = 0.71, 95% CI = 0.56, 0.91). The other variables that showed a statistically significant increase in odds of birth defects in the multivariate final model were: history of miscarriages (OR = 1.68, 95% CI = 1.30, 2.18), family history of birth defects (OR = 3.84, 95% CI = 1.64, 8.96), infection (OR = 1.44, 95% CI = 1.08, 1.91), taking medicine (OR = 1.70, 95% CI = 1.33, 2.18), pesticide exposure (OR = 2.77, 95% CI = 1.71, 4.49), and twin pregnancy (OR = 3.83, 95% CI = 2.14, 6.87).

**Figure 1:**
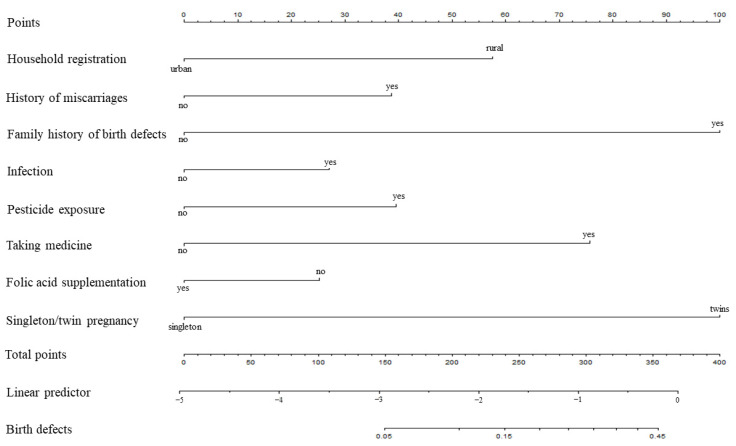
Nomogram for predicting birth defects.

Based on the logistic multivariate regression analysis, the eight independent predictors were included in the prediction model. We then established an individualized nomogram prediction model for the birth defects (Figure 1). The application of the nomogram was structured as follows. Based on the nomogram, we obtained the points corresponding to each prediction indicator, the sum of the points was recorded as the total score, and the predicted risk corresponding to the total score was the probability of birth defects.

**Figure 2:**
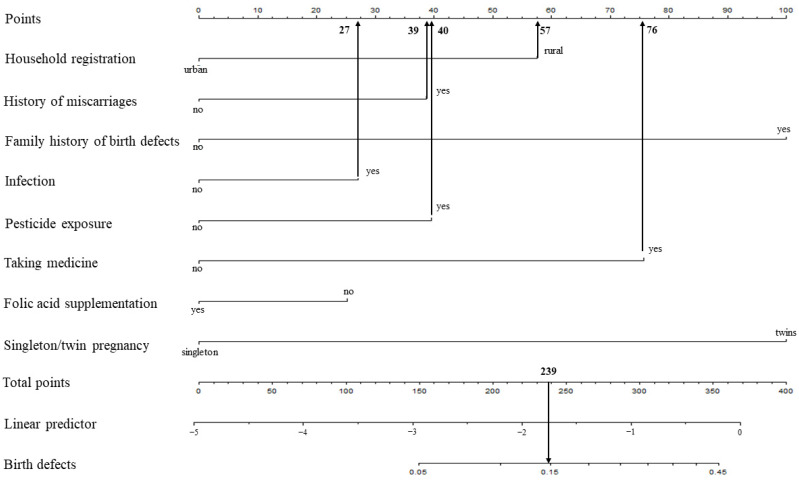
Example prediction nomogram for risk of birth defects.

For example, a pregnant woman living in a rural area (57 points) with a history of miscarriages (39 points), no family history of birth defects (0 points), infection (27 points), pesticide exposure (40 points), taking medicine (76 points), folic acid supplementation (0 points), or single pregnancy (0 points), the cumulative score of the various prediction indicators was 57 +39 + 27 + 40 + 76 = 239, and the corresponding predicted risk of birth defects in her offspring was 0.15 (15%) (Figure 2). 

### 3.3. Nomogram Validation

**Figure 3:**
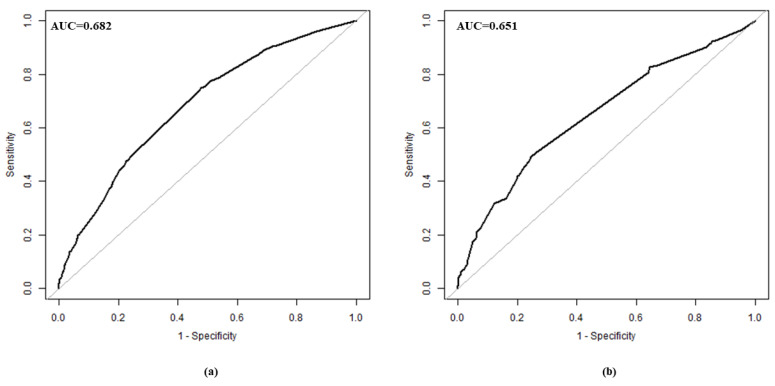
ROC curve in training group (**a**) and validation group (**b**). ROC, receiver operating characteristic.

**Figure 4:**
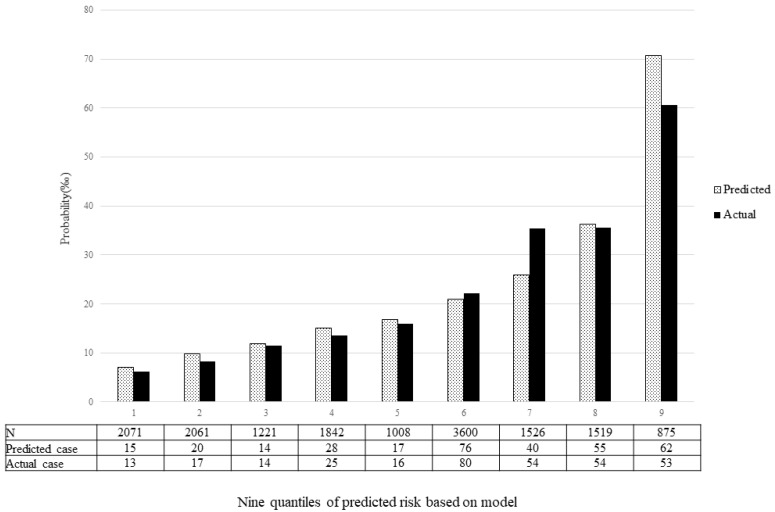
Calibration plot. The *x*-axis represents nine quantiles of predicted risk, and the *y*-axis reveals predicted and actual probability of birth defects.

The validation of the model was based on discrimination and calibration. We drew the ROC curves of the predicted probability and calculated the AUC values in the training and validation group, respectively. The ROC curves were used to compute the AUC values from the models with the eight independent predictors in the nomogram. The AUC values of the training group and validation group were 0.682 (95% CI = 0.653, 0.710) and 0.651 (95% CI = 0.614, 0.689) (Table 4, Figure 3), respectively, suggesting that the nomogram prediction model had a moderate discrimination. The HL χ^2^ statistics was 8.106 (*p* = 0.323), which revealed that the prediction model had good calibration (Figure 4).

## 4. Discussion

A limited number of predictive models for the risk of birth defects have been developed. This study included 29,204 women in Shaanxi, China, which was by far the largest sample used in the development of a risk-prediction nomogram to assess the risk of any type of birth defect in offspring. In our model, the critical predictors of birth defects were household registration, history of miscarriages, family history of birth defects, infection, taking medicine, pesticide exposure, folic acid supplementation, and single/twin pregnancy.

According to the World Health Organization’s estimates, the total prevalence rates of birth defects in developed, middle-income, and low-income countries are 47.2, 55.7, and 64.2 per 1000 live births, respectively. The total prevalence rate of birth defects in China is close to that of middle-income countries [21,22]. Poverty, malnutrition during pregnancy, irrational drug use, poor health care, and a lack of environmental protection lead to the high incidence of birth defects in low- and middle-income countries [8]. Based on China’s Birth Defects Surveillance System, the incidence of birth defects during the perinatal period in China is rising, with 153.23 birth defects per 10,000 in 2011 [6]. Due to the large population base, the total number of new cases of birth defects is extremely high every year in China. Therefore, the primary and secondary prevention of birth defects is an urgent and essential public health task in China.

Our study found that living in urban areas and folic acid supplementation were associated with a decreased risk of birth defects. Based on a nationwide hospital-based registry (the Chinese Birth Defects Monitoring Network), from 2006 to 2008, Li X et al. found that the prevalence ratio of neural tube defects in rural women was much higher than in urban women (21.9 vs. 10.1 per 10,000) [23]. In a previous study, using propensity-score matching, we found that optimal folic acid supplementation was associated with a decreased prevalence of birth defects, especially of the cardiovascular system and nervous system [17]. Our study also found that abortion history, family history of birth defects, infection, taking medicine, pesticide exposure, and twin pregnancy were associated with an increased risk of birth defects. The previous studies confirmed that women with a history of adverse pregnancy, twin pregnancy and adverse environmental and individual exposure are associated with an increased risk of congenital heart defects, spina bifida hypospadias, and other birth defects [19,24,25,26,27,28]. Feng et al. conducted a systematic review and meta-analysis, and found that a history of abortion was associated with a 24% higher risk of congenital heart defects (OR = 1.24, 95% CI = 1.11, 1.38). With a history of spontaneous abortion and induced abortion, the risk of congenital heart defects increased by 18% and 58%, respectively [25]. Based on a population-based case-control study in the USA, Dawson et al. investigated the association between twinning and birth defects. There was a positive relationship between twinning and 29 types of birth defects in the unassisted conception stratum, and cloacal exstrophy and multiple ventricular septal defects showed the largest effects [29].

Other predictive models have been developed for birth defects. Based on epidemiological field data, using logistic regression, Liang Y et al. developed a prediction model that can be used to identify pregnant women who are at high risk of offspring congenital heart defects in Nanchong City, China [30]. Wang JF et al. collected socioeconomic and geographical factors for 7880 live births, and used a support vector machine to develop a prediction model for neural tube defects [31]. Based on a case-control study, Li et al. developed an artificial neural network model that included 15 predictors to select the best model for the prediction of the risk of congenital heart defects in individuals [32]. However, most of these studies were focused on a specific type of birth defect, and were small samples. Compared to prior studies, our study used data from a large birth-defects survey, including 29,204 participants. Additionally, we created a prediction model for any type of birth defect. Thousands of different birth defects affect the structure or function of fetuses. Most of the clinical studies and predictive models were focused on major birth defects, such as congenital heart defects and neural tube defects [30,31,32]. Based on a predictive model for any type of birth defect, we developed a nomogram that can be used as a preliminary screening tool to identify pregnant women at high risk of producing offspring with any birth defects and that can help to guide prenatal management and prevention. Our predictive model provides new possibilities for the prevention of birth defects with low incidence.

The current study has several limitations. Firstly, it was based on a survey database. Both the maternal lifestyle behaviors (maternal smoking, and alcohol consumption) and the data on risk-factor exposure during early pregnancy were obtained through the questionnaire, which will have introduced recall bias. Secondly, some birth defects, especially some genetic diseases, may not have been detected due to the relatively short follow-up of some of the newborns in this study. Thirdly, although we used eight significant factors to establish a prediction model for the risk assessment of the birth defects in the offspring, some variables, such as obesity and gestational diabetes mellitus, which are related to birth defects, were not included in the model because the birth-defect database was limited. Furthermore, the dietary data were collected from only a few of the women in the survey database. Therefore, the nutritional factors, except for the folic acid supplementation, were not included in the prediction model. Therefore, further improvements to the prediction model by adding more prognostic factors are needed in future studies. Additionally, although we used samples from different regions in Shaanxi province to validate the model, we still need evidence from another sample for validation. Lastly, our study was based on the Northwest Chinese population, which should be considered in the extrapolation of the prediction model.

## 5. Conclusions

In summary, we developed and validated a nomogram risk model for any type of birth defect in a Chinese population based on a large birth-defect survey and important modifying factors in pregnant women. This prediction model accurately predicted the birth defects based on the women’s risk-factor exposure in pregnancy. The model can therefore be a potential tool for clinicians to assess the risk of birth defects and promote health education.

## Figures and Tables

**Table 1:** Basic characteristics of training group and validation group (%).

Variables	Training Group (*n* = 15,723)	Validation Group (*n* = 13,481)	χ^2^ Value	*p* Value
Household registration, *n* (%)			1751.258	<0.001
rural	8899 (56.60)	10,738 (79.65)		
urban	6824 (43.40)	2743 (20.35)		
Age, *n* (%)			108.482	<0.001
<30 years	11,634 (73.99)	10,675 (79.19)		
≥30 years	4089 (26.01)	2806 (20.81)		
Years of education, *n* (%)			1280.020	<0.001
<9 years	8233 (52.36)	9810 (72.77)		
≥9 years	7490 (47.64)	3671 (27.23)		
Gravidity, *n* (%)			27.682	<0.001
1	8260 (52.53)	6666 (49.45)		
≥2	7463 (47.47)	6815 (50.55)		
History of preterm birth, *n* (%)			5.300	0.021
no	15,316 (97.41)	13,072 (96.97)		
yes	407 (2.59)	409 (3.03)		
History of miscarriages, *n* (%)			135.328	<0.001
no	13,196 (83.93)	11,951 (88.65)		
yes	2527 (16.07)	1530 (11.35)		
Family history of birth defects, *n* (%)			0.679	0.410
no	15,645 (99.50)	13,423 (99.57)		
yes	78 (0.50)	58 (0.43)		
Infection, *n* (%)			7.721	0.005
no	13,586 (86.41)	11,797 (87.51)		
yes	2137 (13.59)	1684 (12.49)		
Taking medicine			65.867	<0.001
no	12,859 (81.78)	11,503 (85.33)		
yes	2864 (18.22)	1978 (14.67)		
Alcohol drinking, *n* (%)			16.063	<0.001
no	15,579 (99.08)	13,290 (98.58)		
yes	144 (0.92)	191 (1.42)		
Tobacco exposure, *n* (%)			23.359	<0.001
no	6175 (39.27)	5670 (42.06)		
yes	9548 (60.73)	7811 (57.94)		
Pesticide exposure, *n* (%)			49.940	<0.001
no	15,476 (98.43)	13,389 (99.32)		
yes	247 (1.57)	92 (0.68)		
Industrial exposure, *n* (%)			214.208	<0.001
no	10,991 (69.90)	10,447 (77.49)		
yes	4732 (30.10)	3034 (22.51)		
Folic acid supplementation, *n* (%)			313.199	<0.001
no	9295 (59.12)	9316 (69.10)		
yes	6428 (40.88)	4165 (30.90)		
Single/twin pregnancy, *n* (%)			0.351	0.555
singleton	15,539 (98.83)	13,313 (98.75)		
twin	184 (1.17)	168 (1.25)		

**Table 2:** Univariate logistic analysis of factors predicting birth defects in the training group.

Variables	Birth Defects (*n* = 326)	Normal (*n* = 15,397)	OR (95 %CI)	*p* Value
Household registration, *n* (%)				
rural	246 (75.46)	8653 (56.20)	-	
urban	80 (24.54)	6744 (43.80)	0.42 (0.32, 0.54)	<0.001
Age, *n* (%)				
<30 years	229 (70.25)	11,405 (74.07)	-	
≥30 years	97 (29.75)	3992 (25.93)	1.21 (0.95, 1.54)	0.120
Years of education, *n* (%)				
<9 years	213 (65.34)	8020 (52.09)	-	
≥9 years	113 (34.66)	7377 (47.91)	0.58 (0.46, 0.73)	<0.001
Gravidity, *n* (%)				
1	145 (44.48)	8115 (52.71)		
≥2	181 (55.52)	7282 (47.29)	1.39 (1.12, 1.74)	0.003
History of preterm birth, *n* (%)				
no	310 (95.09)	15,006 (97.46)	-	
yes	16 (4.91)	391 (2.54)	1.98 (1.19, 3.31)	0.009
History of miscarriages, *n* (%)				
no	247 (75.77)	12,949 (84.10)	-	
yes	79 (24.23)	2448 (15.90)	1.69 (1.31, 2.19)	<0.001
Family history of birth defects, *n* (%)				
no	320 (98.16)	15,325 (99.53)	-	
yes	6 (1.84)	72 (0.47)	3.99 (1.72, 9.25)	0.001
Infection, *n* (%)				
no	259 (79.45)	13,327 (86.56)	-	
yes	67 (20.55)	2070 (13.44)	1.67 (1.27, 2.19)	<0.001
Taking medicine				
no	225 (69.02)	12,634 (82.05)		
yes	101 (30.98)	2763 (17.95)	2.05 (1.72, 2.61)	<0.001
Alcohol drinking, *n* (%)				
no	321 (98.47)	15,258 (99.10)	-	
yes	5 (1.53)	139 (0.90)	1.71 (0.70, 4.20)	0.242
Tobacco exposure, *n* (%)				
no	109 (33.44)	6066 (39.40)	-	
yes	217 (66.56)	9331 (60.60)	1.29 (1.03, 1.29)	0.030
Pesticide exposure, *n* (%)				
no	306 (93.87)	15,170 (98.53)	-	
yes	20 (6.13)	227 (1.47)	4.37 (2.73, 7.00)	<0.001
Industries exposure, *n* (%)				
no	202 (61.96)	10,789 (70.07)	-	
yes	124 (38.04)	4608 (29.93)	1.44 (1.15, 1.80)	0.002
Folic acid supplementation, *n* (%)				
no	228 (69.94)	9067 (58.89%)	-	
yes	98 (30.06)	6330 (41.11%)	0.62 (0.49, 0.78)	<0.001
Singleton/twin pregnancy, *n* (%)				
singleton	313 (96.01)	15,226 (98.89)	-	
twin	13 (3.99)	171 (1.11)	3.70 (2.08, 6.57)	<0.001

**Table 3:** Multivariate logistic analysis of factors predicting birth defects in the training group.

Variables	B	OR (95% CI)	*p* Value
Household registration			
rural	-	-	
urban	−0.774	0.46 (0.36, 0.60)	<0.001
History of miscarriages			
no	-	-	
yes	0.520	1.68 (1.30, 2.18)	<0.001
Family history of birth defects			
no	-	-	
yes	1.344	3.84 (1.64, 8.96)	0.002
Infection			
no	-	-	
yes	0.363	1.44 (1.08, 1.91)	0.012
Taking medicine			
no	-	-	
yes	0.532	1.70 (1.33, 2.18)	<0.001
Pesticide exposure			
no	-	-	
yes	1.018	2.77 (1.71, 4.49)	<0.001
Folic acid supplementation			
no	-	-	
yes	−0.339	0.71 (0.56, 0.91)	0.006
Single/twin pregnancy			
singleton	-	-	
twin	1.343	3.83 (2.14, 6.87)	<0.001

**Table 4:** The AUCs of the ROC curves for the nomogram and variables from the logistic regression model in the training group and validation group.

Variables	Training Group			Validation Group		
	AUC	95% CI	*p* Value	AUC	95% CI	*p* Value
Nomogram variable	0.682	0.653, 0.710	<0.001	0.651	0.614, 0.689	<0.001
Household registration	0.596	0.567, 0.625	<0.001		-	
History of miscarriages	0.542	0.509, 0.575	0.010		-	
Family history of birth defects	0.507	0.475, 0.539	0.671		-	
Infection	0.536	0.503, 0.569	0.028		-	
Taking medicine	0.565	0.532, 0.599	0.002		-	
Pesticide exposure	0.523	0.490, 0.556	0.149		-	
Folic acid supplementation	0.555	0.525, 0.586	0.001		-	
Single/twin pregnancy	0.514	0.482, 0.547	0.373		-	

## Data Availability

The data presented in this study are available on request from the corresponding author. The data are not publicly available due to project regulations.

## Supplementary Materials

The following supporting information can be downloaded at: https://www.mdpi.com/article/10.3390/ijerph19148584/s1, Table S1: Prevalence of birth defects in the survey (per 10,000). Figure S1: Eligibility assessment with exclusion criteria.
