# Supplementary material for: Risk Assessment for Birth Defects in Offspring of Chinese Pregnant Women
Source: Int J Environ Res Public Health. 2022 Jul 14;19(14):8584. doi: 10.3390/ijerph19148584 (PMC9319985; doi:10.3390/ijerph19148584)
Supplement: Supplementary file 1 [file ijerph-19-08584-s001.zip › ijerph-1751841-supplementary.pdf]

Supplementary Table S1. Prevalence of birth defects in the survey (per 10,000).

	Training group (n=15,723)	Validation group (n=13,481)	Total (n =29,204)	Proportion (n =562)
Cardiovascular system defect	94 (59.79)	91 (67.50)	185 (63.35)	32.92%
Musculoskeletal system	61 (38.80)	40 (29.67)	101 (34.58)	17.97%
Eye, ear, face and neck	46 (29.26)	24 (17.80)	70 (23.97)	12.46%
Oral clefts	15 (9.54)	19 (14.09)	34 (11.64)	6.05%
Digestive system	13 (8.27)	12 (8.90)	25 (8.56)	4.45%
Nervous system	10 (6.36)	10 (7.42)	20 (6.85)	3.56%
Genital organs	10 (6.36)	7 (5.19)	17 (5.82)	3.02%
Respiratory system	8 (5.09)	3 (2.23)	11 (3.77)	1.96%
Urinary system	2 (1.27)	4 (2.97)	6 (2.05)	1.07%
Chromosomal abnormalities	2 (1.27)	0 (0.00)	2 (0.68)	0.36%
Other defects	65 (41.34)	26 (19.29)	91 (31.16)	16.19%
Total birth defects	326 (207.34)	236 (175.060)	562 (192.44)	100%

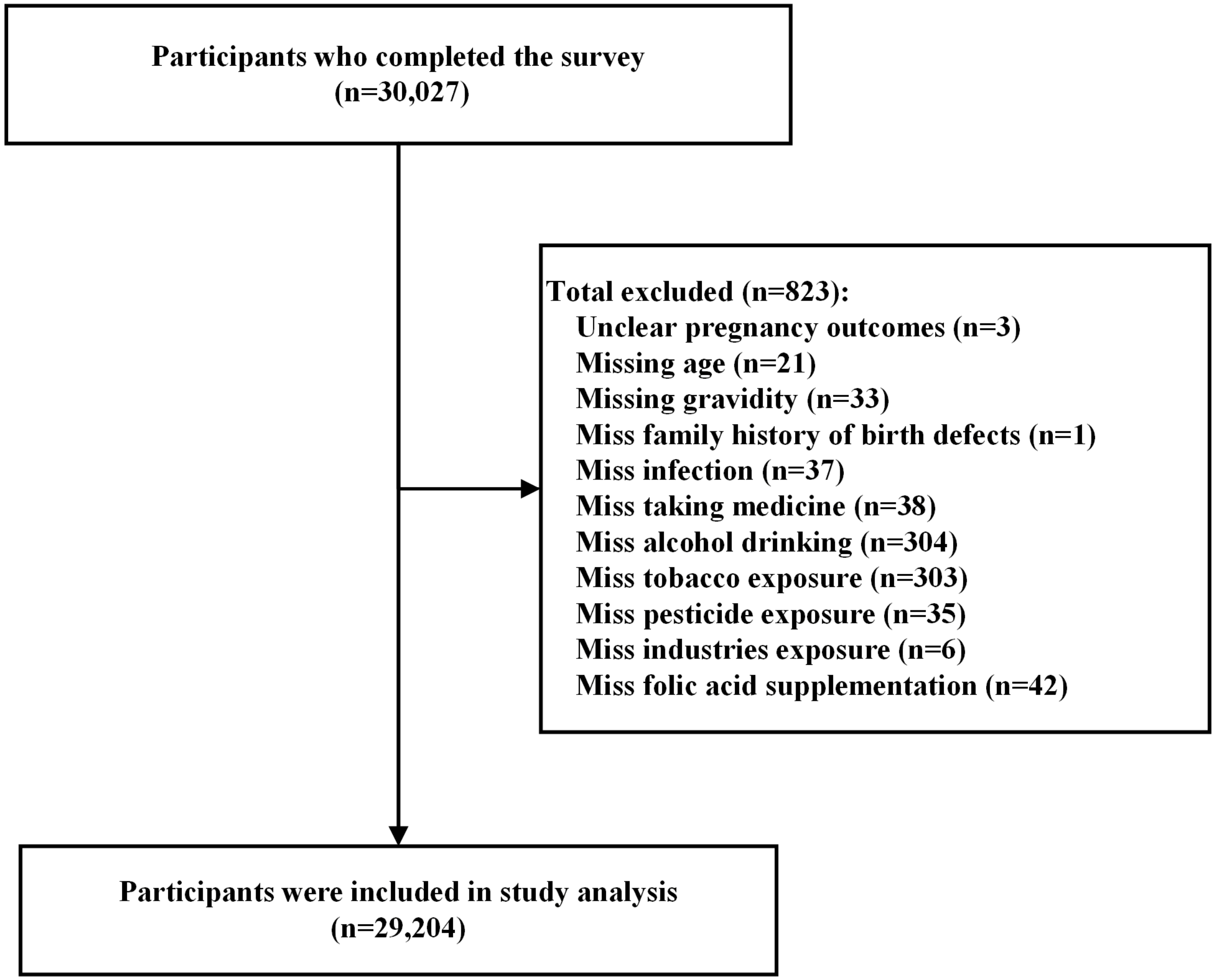

Supplementary Figure S1. Eligibility assessment with exclusion criteria.
